# 

**Published:** 1910-01-22

**Authors:** 


### BOOKS RECEIVED.

MESSRS. LONGMANS, GREEN AND CO.

"Essentials of Chemical Physiology." By W. D. Halliburton, M.D., LL.D., F.R.S.

CHARLES GRIFFIN AND CO., LTD.

"The Year-book of the Scientific and Learned Societies of Great Britain and Ireland."

CASSELL AND CO.

"Hypnotism and Treatment by Suggestion." By J. Milne Bramwell, M.B., C.M.

"Serums, Vaccines and Toxines in Treatment and Diagnosis." By W. C. Bosanquet, M.A., etc., and J. W. H. Eyre, M.D., etc.

"Syphilis." By Sir Jonathan Hutchinson, F.R.S., LL.D., F.R.C.S.

REBMAN, LTD.

"A Short Handbook of Cosmetics." By Dr. Max Joseph.

CHATTO AND WINDUS.

"London Charities." Edited by John Lane.

E. AND S. LIVINGSTONE.

"Public Health." Cathecism series; Parts I., II., III., IV., and V.

HENRY KIMPTON.

"The Care of Children from Babyhood to Adolescence." By B. Myers, M.D., etc.

HENRY FROWDE AND HODDER AND STOUGHTON.

"Male Diseases in General Practice." By Edred M. Corner, M.A.

THE AMBROSE CO., LTD.

"Religion and Insanity." By E. Williams, L.R.C.P., L.R.C.S., L.S.A.

"Spiritualism and Insanity." By Dr. C. Williams.

JOHN BALE, SONS AND DANIELSSON, LTD.

"The Stomach, Intestines, and Pancreas." By W. C. Bosanquet, M.A., etc., and H. S. Clogg, M.A., etc.

KING, SELL AND OLDING, LTD.

"The Science Year-book," 1910.

### Gifts and Bequests.

The Mercers' Company have sent ten guineas to the British Lying-in Hospital, Endell Street, W.C.

The Treasurer of Bart's (Lord Sandhurst) acknowledges further contributions in response to the Lord Mayor's appeal issued in October:—London City and Midland Bank, Ltd., £105; "R. D. W.," £100; Tallow Chandlers' Company, £10 10s.; Mrs. Caleb Trapnell, £5; Miss M. S. Farrer, £5; Mr. Mark Fenwick, £5; Mrs. Dawson, £5.

Miss Annie Ellin Sandars has bequeathed £100 to the Mold Cottage Hospital.

Under the will of Miss Ellen Morrison, of Basildon Park, Pangbourne, which was made only two days before her death, the following bequests to charities have been made:—£5,000 to the Royal Berks County Hospital, Reading; £2,000 to the King Edward VII. Hospital Fund. Miss Morrison's estate has now been valued, so far as can at present be ascertained, at £975,000.

Among the latest contributions to King Edward's Hospital Fund for London is the Queen's annual subscription of £25.

Mr. Alfred George Beebe has left £1,000 to Guy's Hospital for the endowment of a bed, £500 each to the Alexandra Hospital for Children with Hip Disease, the Victoria Hospital for Children for the endowment of beds, and £100 to the Royal Hospital for Incurables.

Miss Elizabeth Louisa Vance has left £100 each to the Cancer Hospital, Fulham, the Brompton Hospital for Consumption and Diseases of the Chest, the Royal Hospital for Incurables.

The Wood Green, Hornsey, and Southgate Passmore Edwards Hospital will receive a legacy of £2,000 under the will of the late Mr. Leggatt, of Hornsey.

Mrs. Elizabeth Mary Walshe has left £200 to St. Vincent's Hospital, Dublin.

Mrs. Sarah Smith, of Rochester, has left £200 to St. Bartholomew's Hospital, Rochester.

### Appointments.

Professor G. Sims Woodhead has been appointed Vice-President of the Society of Medical Phonographers.

Dr. John T. Maclachlan, M.D., has been appointed assistant physician to the Royal Infirmary, Glasgow.

The following Public Health appointments as Medical Officers of Health have been made:—Mr. J. Craig, to Church Urban District; Mr. S. L. Melville, to Middlewich Urban District; Mr. T. Aubrey, to Warmley Rural District.

### Obituary.

The death took place on January 10 of Mr. Septimus J. Lee, L.R.C.P. (Lond.), M.R.C.S. (Eng.), of Brampton, Cumberland. He had been resident medical officer at the Hospital for Epilepsy and Paralysis, Maida Vale, since April 1909. Mr. Lee studied at Edinburgh and at Middlesex Hospital, and was appointed to the Hospital for Epilepsy as soon as he had qualified last year.

The *Gazette* of January 14 published an Order in Council declaring that the second part of the Medical Act, 1886, shall be deemed as from the date of the Order to apply to the province of Prince Edward Island in the Dominion of Canada.

The following lectures, inspections, and demonstrations, among others, will be given under the auspices of the Royal Sanitary Institute, 90 Buckingham Palace Road :—

Wednesday, February 16 : Demonstration of book-keeping, as carried out in a sanitary inspector's office, at the Public Health Office, Town Hall, Upper Street, Islington, N., at 6 p.m., by James R. Leggatt, superintendent, Public Health Department, Borough of Islington. Saturday, February 26 : Inspection and demonstration at the Battersea Disinfection Station, Mortuary and Shelter, at 2.15 p.m. Conducted by G. Quin Lennane, F.R.C.S., L.R.C.P., D.P.H., M.O.H., Battersea.

Saturday, March 5 : Inspection and demonstration at Hampton Sewage Disposal Works, at 2.30 p.m. Conducted by S. H. Chambers, Surveyor to District Council. Wednesday, March 16 : Demonstration on baths and lavatories in the Parkes Museum, at 6 p.m. Friday, March 18 : Demonstration on waste preventers and water closets in the Parkes Museum, at 6 p.m. Saturday, March 19 : Inspection and demonstration at the sewage and destructor works, Ealing, at 2.15 p.m. Conducted by Charles Jones, M.Inst.C.E., Borough Engineer and Surveyor. Tuesday, March 22 : Demonstration on pipes, joints, etc., and drain-testing appliances in the Parkes Museum, at 6 p.m. Wednesday, March 23 : Demonstration on water supply in the Parkes Museum, at 6 p.m.

### THE QUESTION BOX.

**SPECIAL NOTICE.**—Questions of interest affecting the honorary medical staff and hospital administration in all departments are invited. When any point arises we hope our readers will formulate it in a question and so co-operate to make this column of real value and interest. In this way the experience of the best-managed hospitals should be made helpful to the whole hospital world. We shall welcome the contribution of both questions and answers.

N.B.—The publication of an answer in this column does not necessarily preclude the publication of subsequent answers to the same question, for a question may involve points which require to be threshed out and fully discussed. Answers, if published, will be paid for at scale rates.

### WANTED, A SYSTEM FOR FILING.

Question: What is the best system for filing records, documents, and letters suitable for a large hospital?

### A Coventry Practice.

By Mr. J. A. Rudd, Secretary Coventry and Warwickshire Hospital.

We have recently adopted at the Coventry and Warwickshire Hospital the card index system for registering both in- and out-patients. The system is most elastic and can be applied for filing and recording almost anything. By its use the history of a patient can be immediately obtained, whether he was discharged only yesterday or ten years ago. Each patient's name is indexed on a small card (5 in. by 3in.), and which bears his name and address, register number, etc. This card is then filed alphabetically in a suitable case or drawer. When the patient is discharged his history sheet is sent to the Secretary's office, where the register number is filled in and the various papers bound together and filed in numerical order. The index card is transferred to a transfer case, which becomes the permanent index; the current index thus contains only the cards of patients in the hospital, and is a check on the daily count, etc. To suit the requirements of our hospital I have adopted my own design of a combined history and temperature chart. The system has also been applied to out-patients in much the same manner (the prescription and diagnosis sheet taking the place of the "history-chart" sheet of the in-patient) and is worked by the O.P. porter. In a large hospital it would no doubt be done by the registrar or inquiry officer.

I would warn all secretaries intending to adopt this system not to be persuaded into buying unlimited filing cabinets sold by card index firms. I regard these as so much furniture, cumbering valuable space. We do not use a cabinet for in-patients; Shannon files are cheap and good, and answer the purpose better than a cabinet. We use a two-drawer cabinet for out-patients; the papers for these patients when discharged are filed away in shelves in an upright position.

Before adopting the system I visited the Eye Hospital, Birmingham, where it has been in operation for some time, and take this opportunity of recording my thanks to Mr. Mason, the secretary, for much valuable information obtained. Our Chairman, and the officials at his works, gave much valuable assistance in inaugurating the system.

### FIRE IN HOSPITALS.

### WHAT TO DO IN CASE OF FIRE.

### A Southport Practice.

Mr. J. H. Shaw, Secretary, Southport Infirmary, sends the following directions in force at this institution :—

(1) Close all doors and windows in the vicinity of the outbreak. (2) Fetch nearest "Patrol" extinguisher, turn same upside down, and direct chemical stream on to the fire. (3) Telephone to police station (No. 69) for fire brigade. (4) Summon the staff by sounding the fire gong. The Engineer, when present, is appointed to direct the operations until the arrival of the fire brigade. (5) Turn off gas at the meters. (6) Collect "Patrol" extinguishers throughout the building, and all available buckets, and use same until the firemen arrive. (7) Couple the garden hose to any tap near the seat of the fire, and direct water on to same. (8) If serious, prepare to remove the patients to a place of safety. Unlock the mortuary and the old mortuary, which might be temporarily utilised. Get out the fire ladder from the old mortuary. (9) Escape from the nurses' quarters may be effected—(*a*) by the staircase; (*b*) by the back bedroom windows; (*c*) by the fire ladder; or these failing (*d*) by one of the ropes fixed at each end of the corridor. (10) Escape from the servants' quarters may be effected—(*a*) by the staircase; (*b*) by the fire ladder; or (*c*) by the window of bedroom No. 5 on to the larder roof. (11) In approaching dense smoke, if possible cover mouth and nose with a wet cloth.

### A Practical Experience.

By Mr. C. S. Risbee, Secretary-Superintendent, Northampton General Hospital.

In The Hospital of December 18, 1909, in the report of the committee appointed by his Royal Highness the Prince of Wales, the question of alarming patients in case of fire is dealt with. Some people here objected to our putting up a fire bell on that ground, but by having frequent drills just the opposite effect is produced. When fire broke out here many of the patients and members of the household knew nothing about it until the Fire Brigade had arrived and all immediate danger was over; and I can assure you there was not the slightest scare of any kind. You will perhaps say what about the night? Even then I do not think there would be much alarm, for the nurses would calm the patients by telling them that it was probably only drill. We have had one or two night drills, though not in the middle of the night.

### THE COMMITTEE OF MANAGEMENT AND THE HONORARY MEDICAL STAFF.

### THE METHOD OF REPRESENTATION.

Question: If it is desirable that the Honorary Medical Staff should be members of the Committee of Management, should they all be members *ex officio*, or only some of them? If the latter, how many medical representatives should there be on the lay Committee, and how should they be elected?

### A Birmingham View.

Answer: Limited representation with continunity.
By Mr. Howard J. Collins, House Governor, Birmingham General Hospital.

I think that all the full physicians and surgeons should be members of the General Committee or Board of Management, with one representative of the Special Departments (but the total number should be limited so that it does not exceed one-third of the lay members). I think there should also be two representatives (a physician and a surgeon) on the House Committee (or weekly managing body). They should be elected by the Medical Committee for a period of two years, but should be so appointed that they retire in alternate years, so that there is always one who has been on for twelve months when a new member is appointed. By this means continuity of representation is ensured and unnecessary discussion is avoided.

THE

### GRADUATES' MEDICAL SCHOOLS.

DIARY FOR THE WEEK, JANUARY 24 to 28.

### THE BROMPTON HOSPITAL FOR CONSUMPTION AND DISEASES OF THE CHEST, Fulham Road, S.W.

**At 4 p.m.—Jan. 26,** Dr. Habershon, **The Examination of the Chest.**

### ST. JOHN'S HOSPITAL FOR DISEASES OF THE SKIN, Leicester Square, W.C.

**At 6 p.m.—Jan. 27,** Chesterfield Lecture: **Seborrhœa and Psoriasis.**

### MEDICAL GRADUATES' COLLEGE AND POLYCLINIC, 22 Chenies Street, W.C.

**At 4 p.m.—Jan. 24,** Dr. T. Colcott Fox, **Clinique (Skin).**

**At 5.15 p.m.—Jan. 24,** Dr. Theo. Hyslop, **The Examination of Insane Criminals.**

**At 4 p.m.—Jan. 25,** Dr. Alex. Morison, **Clinique (Medical).**

**At 5.15 p.m.—Jan. 25,** Mr. Edred M. Corner, **The Treatment of Septic Conditions.**

**At 4 p.m.—Jan. 26,** Mr. T. H. Openshaw, **Clinique (Surgical).**

**At 5.15 p.m.—Jan. 26,** Dr. R. A. Gibbons, **On Dysmenorrhœa.**

**At 4 p.m.—Jan. 27,** Sir Jonathan Hutchinson, **Clinique (Surgical).**

**At 5.15 p.m.—Jan. 27,** Dr. James Taylor, **Eye Symptoms and Signs in Nervous Diseases.**

**At 4 p.m.—Jan. 28,** Mr. R. E. Bickerton, **Clinique (Eye).**

### THE POST-GRADUATE COLLEGE, West London Hospital, Hammersmith, W.

**At 10 a.m.—Jan 24 and 27, Demonstration of Cases by Surgical Registrar.**

**At 10 a.m.—Jan. 28, Demonstration of Cases by Medical Registrar.**

**At 12 noon.—Jan. 27,** Dr. Bernstein, **Pathological Demonstration.**

**At 12.15 p.m.—Jan. 26,** Dr. Grainger Stewart, **Practical Medicine.**

**At 5 p.m.—Jan. 24,** Mr. Baldwin, **Practical Surgery.**

**At 5 p.m.—Jan. 25,** Dr. Pritchard, **Clinical Pathology.**

**At 5 p.m.—Jan. 26,** Dr. Beddard, **Medicine: Lecture II.**

**At 5 p.m.—Jan. 27,** Dr. Cole, **Adolescent Insanity.**

**At 5 p.m.—Jan. 28,** Dr. Morton, **X-Ray Examination of the Digestive System.**

### ROYAL SOCIETY OF MEDICINE, 20 Hanover Square, W.

**At 8 p.m.—Jan. 24, Odontological Section:**

*Papers.*—(Adjourned from November meeting) Some observations on the bacteriology of pyorrhœa alveolaris, and the results of treatment by bacterial vaccines, Dr. Eyre and Mr. J. Lewin Payne. Mr. Payne will give his supplementary remarks. Mr. Kenneth Goadby: The vaccine treatment of early cases of pyorrhœa alveolaris. The discussions on both papers will be taken simultaneously and opened by Mr. Goadby.

**At 5.30 p.m.—Jan. 25, Medical Section:**

*Paper.*—Dr. Llewellyn Phillips: The treatment of tetanus by the intraspinal injection of a solution of magnesium sulphate; with cases.

**At 5.30 p.m.—Jan. 27, Balneological and Pharmacological Section:**

*Paper.*—Dr. Ackerley: Saline waters, and the use and abuse of common salt. Members of the Medical and Therapeutical sections are specially invited to attend this meeting.

**At 8.30 p.m.—Jan. 27, Neurological Section:**

A discussion on "Syphilitic and metasyphilitic diseases of the nervous system" will be opened by Dr. Frederick Mott, F.R.S. Dr. Henry Head, F.R.S., will continue the discussion. Those gentlemen desirous of joining in the discussion will kindly notify Dr. W. Harris, 73 Wimpole Street, W. There will be an exhibition of microscopic sections and diagrams bearing on the subject, by Drs. Mott and Gordon Holmes.

**At 4.30 p.m.—Jan. 28, Section for the Study of Disease in Children:**

*Cases.*—Dr. Frew (for Dr. F. E. Batten): Meningococcal meningitis treated by meningococcal serum (Lister) with rapid improvement. Mr. Sydney Stephenson and Dr. Eric Pritchard: Basedow's disease in a lad of nine years. Dr. F. Parkes Weber: (1) Splenomegaly and hydrocephalus; (2) family amaurotic idiocy without characteristic ophthalmoscopic signs; (3) internal hydrocephalus and amaurosis without definite ophthalmoscopic changes following symptoms of posterior basic meningitis. Dr. O. K. Williamson: Spastic diplegia. Mr. Douglas Drew: Separation of lower epiphysis of femur with displacement forward on to the shaft. Mr. J. M. G. Swainson: A child from whom a strangulated ovarian cyst was removed. Dr. Vincent Dickinson: Enlarged spleen. Mr. Bishop Harman: (1) Abnormal congenital pigmentation of one eye; (2) two cases of familial discoid or Coppock cataract.

*Specimens.*—Drs. H. D. Rolleston and A. C. D. Firth: Hemiatrophy of brain in an infant. Dr. J. D. Rolleston: Diphtheria of the fauces and ano-genital region in an infant. Dr. Forsyth: Specimen from a case of congenital œdema with dilatation of the abdominal lymphatics. Drs. E. I. Spriggs and H. F. L. Hugo: *X*-ray photographs from a case of constipation. Dr. A. C. D. Firth: Prolapse of intestine through ectopia vesicæ.

*Papers.*—Dr. A. E. Russell: A case of cyclic vomiting in a child, associated with hypertrophic stenosis of the pylorus. Dr. Donald Carter: Infant feeding.

**At 8.30 p.m.—Jan. 28, Epidemiological Section:**

*Paper.*—Dr. F. Graham Crookshank: The control of scarlet fever.

MR. JOHN BURNS has appointed Dr. Eastwood, a Royal Commissioner on Tuberculosis, a Medical Inspector of the Board, to undertake pathological investigations. The object is to apply to public health work the results of the Tuberculosis Commission, and to free foods from infection by this disease. The new work will include inquiry as to the existing pathological methods of diagnosis, and should encourage their standardisation.

THE HOSPITAL. JANUARY 22, 1910.

*Name* ..................................

*Address* ..................................

..................................

**This Coupon must accompany manuscript or contributions intended for THE HOSPITAL.**

